# Postoperative anatomical and functional outcomes of different stages of high myopia macular hole

**DOI:** 10.1186/s12886-015-0098-8

**Published:** 2015-08-07

**Authors:** Qing Shao, Huijuan Xia, Florian M. A. Heussen, Yanling Ouyang, Xiaodong Sun, Ying Fan

**Affiliations:** Department of Ophthalmology, Shanghai First People’s Hospital, Haining Road 100, 200080 Shanghai, China; Department of Ophthalmology, Charité-Universitätsmedizin, Berlin, Germany; Department of Ophthalmology, The Royal Liverpool University Hospital, Liverpool, UK

## Abstract

**Background:**

Recently it was suggested that high myopia macular holes (HMMH) and macular holes accompanied by retinal detachment occur in the advanced stages of myopia traction maculopathy (MTM), while macular retinoschisis, shallow retinal detachment without holes, and lamellar macular holes occur in the early stages of MTM. Complete vitreous cortex removal associated with internal limiting membrane peeling is now widely used to treat HMMH. However, it remains uncertain at what HMMH stage patients would benefit most from surgical intervention. Our study was aimed to evaluate the postoperative anatomical changes and functional outcomes of high myopia macular holes (HMMH).

**Methods:**

Patients were retrospectively collected between March 2009 and August 2011. Before and 1st, 3rd, and 9th month after 23G pars plana vitrectomy, all patients underwent a complete ophthalmologic examination, spectral domain optical coherence tomography (SD-OCT) and MP-1. At each follow-up, best-corrected visual acuity (BCVA), photoreceptor inner and outer segments (IS/OS) defects, and retinal sensitivity (RS) were investigated. According to different preoperative macular hole morphologies, patients were divided into three groups: Group 1, macular hole with epiretinal membrane (ERM) traction and macular retinoschisis; Group 2, full-thickness macular hole (FTMH); Group 3, FTMH with subretinal fluid.

**Results:**

43 eyes from 43 patients met the inclusion criteria. The mean age was 60 years. BCVA and RS were significantly improved after vitrectomy; the mean IS/OS defect was significantly reduced. At 9 postoperative months, 11 of 43 (25.6 %) eyes achieved IS/OS junction integrity; 9 of these 11 (81.8 %) eyes belonged to Group 1, 2 (18.2 %) belonged to Group 2.

**Conclusions:**

Pars plana vitrectomy combined with ILM peeling and gas tamponade results in limited functional outcomes in patients with HMMH. The appearance of subretinal fluid indicates a worse prognosis for surgical intervention.

## Background

Retinopathy in pathological myopia is a major cause of visual impairment, and long axial lengths and posterior staphyloma eventually lead to myopia traction maculopathy (MTM) [1]. The term MTM was first used in 2004 to describe a series of posterior pole pathologies in cases of high myopia, such as macular retinoschisis, shallow retinal detachment without retinal holes, lamellar macular holes, and macular holes, with or without retinal detachment. In 1999, Takano and Kishi speculated that the formation of high myopia macular holes (HMMH) resulted from macular retinoschisis or shallow retinal detachment without holes [2]. Panozzo and Mercanti [3] supported this notion and suggested that HMMH and macular holes accompanied by retinal detachment occur in the advanced stages of MTM, while macular retinoschisis, shallow retinal detachment without holes, and lamellar macular holes occur in the early stages of MTM. This hypothesis has been confirmed by several other groups [4–8].

Complete vitreous cortex removal associated with internal limiting membrane peeling is now widely used to treat HMMH. However, it remains uncertain at what HMMH stage patients would benefit most from surgical intervention.

In this study, we used spectral domain optical coherence tomography (SD-OCT) and microperimetry pre- and postoperatively in patients diagnosed with HMMH to observe and analyze the retinal microstructure and functional recovery.

## Materials and methods

### Data collection

Patients with a diagnosis of HMMH were retrospectively collected from the Department of Ophthalmology at Shanghai First People’s Hospital, Shanghai Jiaotong University between March 2009 and August 2011. All patients underwent a standard ophthalmologic examination preoperatively and at 1 month, 3 months, and 9 months after pars plana vitrectomy, including volume OCT scanning with an SD-OCT (Spectralis, Heidelberg Engineering, Heidelberg, Germany) and the MP-1 microperimeter (Nidek Technologies, Padua, Italy). The imaging protocols used are described below. Patient information, such as age, sex, lens status, axial length, history of ophthalmic diseases or surgeries, and ophthalmic diagnoses, was also collected. The following inclusion criteria were applied: subjective visual symptoms; high myopia (> − 6.00 diopters), with an axial length >26.00 mm; and a full-thickness macular hole (FTMH) as confirmed and documented by OCT examination. Patients with diabetes, hypertension, eye surgery within 1 year, one or several peripheral retinal holes or retinal detachment were excluded. Best-corrected visual acuity (BCVA) was measured using E-VA charts, and the results were converted to logMAR units. Approval for data collection and analysis was obtained from the institutional review board of the Shanghai First People’s Hospital. Written consent was obtained from all patients. The research adhered to the tenets set forth in the Declaration of Helsinki.

### SD-OCT

**Fig. 1:**
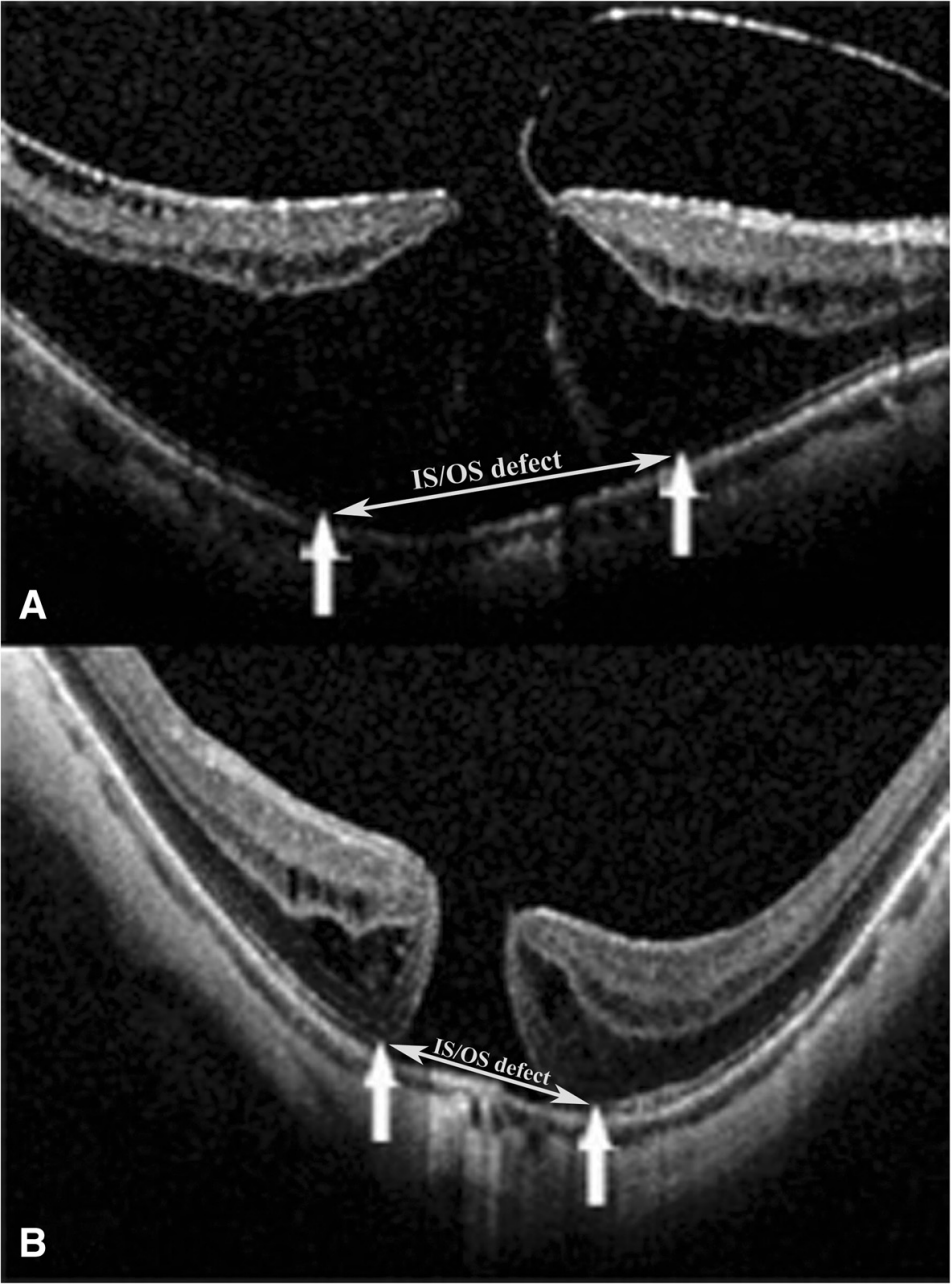
**a** and **b**: Measurements of foveal microstructure on SD-OCT images. The distance between two one-way arrows was measured as IS/OS defect (see two-way arrow). **a** is a preoperative image from Group 1, which showed a macular hole with ERM traction, macular retinoschisis; **b** is FTMH image before surgery in Group 2

All OCT scans were performed with the Spectralis OCT (Heidelberg Engineering, Germany). The scan protocol consisted of 19-line scans centered on the fovea covering 30° radial, with a line spacing of 350 μm between the scans. Active eye tracking and line averaging of 10 scans per line (ART 10) were used. The IS/OS defect was measured by OCT. Only gradable records were used in the study. Two graders (Q.S., Y.F.) independently evaluated each OCT image set (Fig. 1).

### Microperimetry

The retinal sensitivity was measured using the MP1 (Nidek technology, Padua, Italy).

**Fig. 2:**
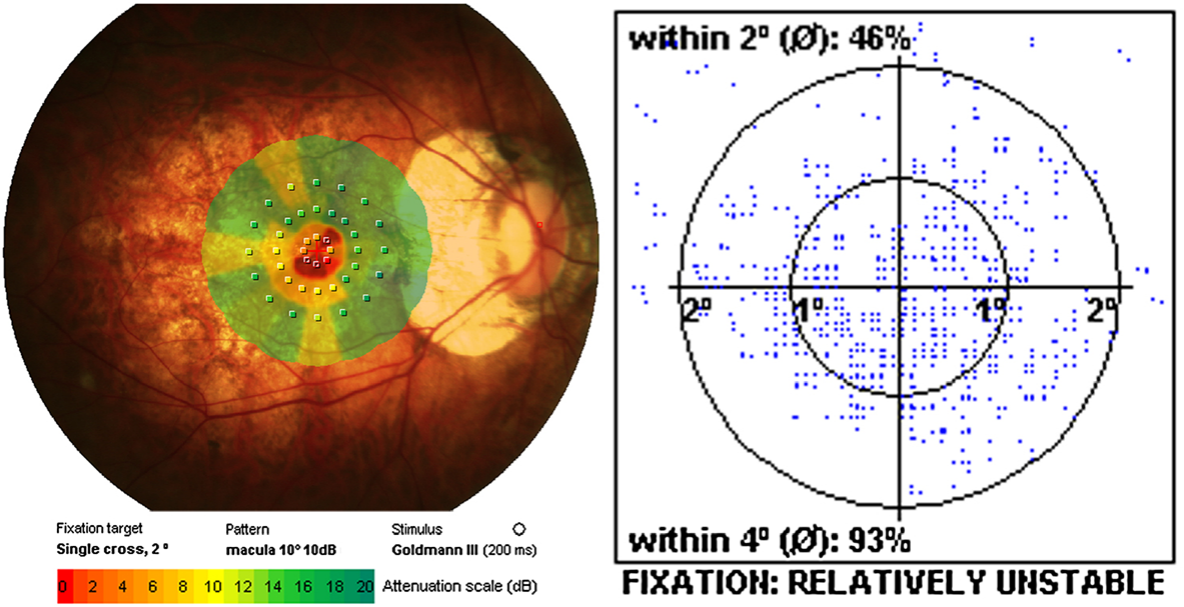
A macular retinal sensitivity map overlaid a fundus color photo which was measured by MP1. The test area was macular 10 degrees. 40 stimulate points were recorded. The luminance range was 0–20 dB. The maximum brightness was 127 cd/m2, minimum brightness was 1.27 cd/m2, and stimulus duration was 200 ms. A staircase strategy of 4-2-1 pattern was used. The average of 40 points retinal sensitivity was calculated

The eye-tracking system corrected eye movements automatically and obtained images with an infrared camera. The test area comprised the macular 10° and 40 stimulus locations within this field. A white background, with a red single cross as a fixation target and Goldmann III white spot stimuli, were used. The luminance range was 0–20 dB. The maximum brightness was 127 cd/m [2], the minimum brightness was 1.27 cd/m [2], and the stimulus duration was 200 ms. A staircase strategy of the 4-2-1 pattern was used. The retinal sensitivity threshold map was automatically registered to the color fundus photo, and the overall average retinal sensitivity was calculated (Fig. 2).

### Groupings

**Fig. 3:**
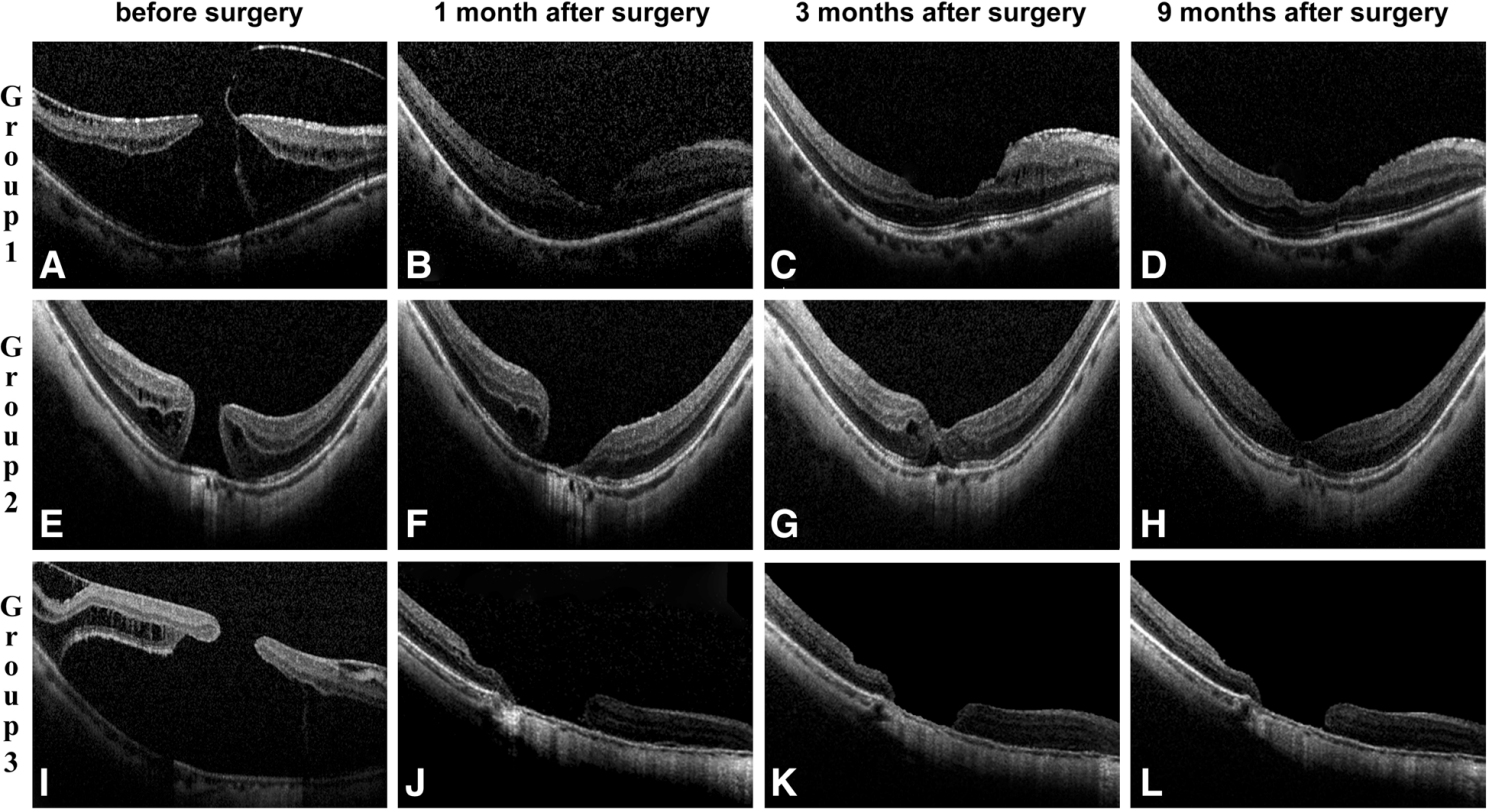
**a**-**l**: Macular microstructures on SD-OCT B-scans before and 1 month, 3 months, 9 months after surgery. **a**-**d**: OCT B-scans from the right eye of a 58 year-old woman in Group 1 before and 1 month, 3 months, 9 months after surgery. The IS/OS defect before and 1 month, 3 months, 9 months after surgery were 1717, 1364, 0, 0 μm, the BCVA were 0.70, 0.52, 0.30, 0.22 logMAR, and the RS were 15.7, 16.8, 17.3, 18.1 dB. **e**-**h**: OCT B-scans from the left eye of a 55 year-old woman in Group 2 before and 1 month, 3 months, 9 months after surgery. The IS/OS defect before and 1 month, 3 months, 9 months after surgery were 1329, 1129, 871, 94 μm, the BCVA were 1, 0.52, 0.30, 0.30 logMAR, and the RS were 10.8, 12.5, 13.5, 15.1 dB. **i**-**l**: OCT B-scans from the right eye of a 54 year-old woman in Group 3 before and 1 month, 3 months, 9 months after surgery. The IS/OS defect before and 1 month, 3 months, 9 months after surgery were 4011, 2635, 2363, 2388 μm, the BCVA were 1.52, 1, 1, 1 logMAR, and the RS were 10.4, 12.6, 12.9, 12.7 dB

Based on the OCT images, the patients were divided into three groups: [9, 10] Group 1, macular hole with epiretinal membrane (ERM) traction, macular retinoschisis; Group 2, FTMH without ERM and subretinal fluid; and Group 3, FTMH with subretinal fluid, with or without ERM (Fig. 3).

### Surgical approach

All patients underwent transconjunctival 23G pars plana vitrectomy. The procedures for 7 eyes were combined with phacoemulsification and intraocular lens implantation in the same surgery. All surgeries were performed by a single surgeon (Y.F.). To stain the internal limiting membrane (ILM), 0.05 % indocyanine green (ICG) was applied. A 15 % perfluoropropane (C3F8) gas tamponade was used at the completion of vitrectomy. The patients were required to maintain a prone position for 7 to 15 days. Eyes with silicone oil tamponade were not included.

### Statistical methods

Data were analyzed using frequency and descriptive statistics. Shapiro-Wilk test was used to assess the normal distribution of the quantitative parameters in this study. To compare differences in pre- and postoperative IS/OS defect, BCVA and RS in each group, Student paired *t*-test was used. Multiple linear regression analysis was used to evaluate correlation between IS/OS defect before surgery and BCVA, RS before and after surgery. Statistical analysis was performed using commercially available SPSS software (Version 19.0, SPSS Inc., Chicago, IL, USA). A two-sided p value of <0.05 was considered to be statistically significant.

## Results

### Demographic characteristics

A total of 48 eyes from 48 patients met the inclusion criteria. Because 5 eyes were lost to follow-up, a total of 43 eyes were included in this study. At month 1, 43 eyes were included. No eyes underwent a second surgery. Twelve eyes were pseudophakic before entering the study, and 7 eyes underwent combined procedures with phacoemulsification and intraocular lens implantation at the initial surgery. The between-group differences regarding age, eye, gender, axial length and cataract surgery were not significant (Table 1). The preoperative parameters regarding age, eye, gender, axial length and cataract surgery had no significant effect in the surgical outcomes (Table 3).

**Table 1:** Comparison of Demographic Characteristics of Each Group

	Group1	Group2	Group3	p value
Number of eyes	17	12	14	--
Age (mean ± SD), years	60.24 ± 6.04	59.33 ± 7.66	60.29 ± 9.24	0.72*
Eye (right)	8	5	10	0.25^a^
Gender (women)	12	11	10	0.35^a^
Axial length, mm	28.03 ± 0.85	27.95 ± 1.11	28.30 ± 1.30	0.85*
Cataract surgery				
Before the initial surgery	5	3	4	0.96^a^
At the initial surgery	3	2	2	0.95^a^
Pseudophakic eyes				
1 month after surgery	8(47 %)	5(42 %)	6(43 %)	0.33^a^
3 months after surgery	8(47 %)	6(50 %)	9(64 %)	0.27^a^
9 months after surgery	12(71 %)	8(67 %)	11(79 %)	0.24^a^
Preoperative parameters				
IS/OS defect, μm	986.16 ± 823.47	2225.49 ± 758.38	4689.08 ± 978.69	<0.0001*
BCVA, LogMAR	0.71 ± 0.33	0.97 ± 0.21	1.65 ± 0.44	<0.0001*
RS, dB	16.27 ± 1.17	13.78 ± 2.67	9.07 ± 2.07	<0.0001*

*IS/OS* photoreceptor inner segment and outer segment, *BCVA* best-corrected visual acuity, *logMAR* logarithm of minimal angle of resolution, *RS* retinal sensitivity. P value represents the difference between groups. P < 0.05 was considered statistically significant. *Kruskal-Wallis test. ^a^Fisher’s exact probability test

### Anatomical results

The mean IS/OS defect of all patients measured by OCT was 2537.62 ± 279.75 μm before surgery and 1120.11 ± 167.60 μm at 9 months after surgery (p < 0.0001). Moreover, the mean IS/OS defect after surgery decreased significantly from follow-up to follow-up in each group. The mean IS/OS defect of Group 1 before surgery was 986.16 ± 823.47 μm, and this value decreased to 318.34 ± 481.72 μm at 9 months after surgery (p < 0.0001). In Group 2, the IS/OS defects before surgery and 9 months after surgery were 2225.49 ± 758.38 μm and 811.76 ± 465.70 μm (p < 0.0001), respectively. The means for Group 3 were 4689.08 ± 978.69 μm before surgery and 2357.98 ± 924.54 μm 9 months after surgery (p < 0.0001) (Table 2).

**Table 2:** IS/OS defect, BCVA and RS Outcomes of 3 Groups Before and After Surgery

Outcomes Parameter	Before surgery	1 month after surgery		3 months after surgery		9 months after surgery	
	Mean ± SD	Mean ± SD	t, p Value*	Mean ± SD	t, p Value*	Mean ± SD	t, p Value*
Group1 (17 eyes)							
IS/OS defect (μm)	986.16 ± 823.47	593.08 ± 697.79	5.02, < 0.0001	415.23 ± 561.58	5.25, < 0.0001	318.34 ± 481.72	5.08, < 0.0001
BCVA (logMAR)	0.71 ± 0.33	0.49 ± 0.25	4.41, 0.0004	0.45 ± 0.25	5.47, < 0.0001	0.43 ± 0.24	5.76, < 0.0001
RS (dB)	16.27 ± 1.17	17.23 ± 1.09	8.35, < 0.0001	17.47 ± 1.12	8.68, < 0.0001	17.62 ± 1.17	8.95, < 0.0001
Group2 (12 eyes)							
IS/OS defect (μm)	2225.49 ± 758.38	1484.31 ± 423.94	5.99, < 0.0001	1097.06 ± 450.65	9.56, < 0.0001	811.76 ± 465.70	10.83, < 0.0001
BCVA (LogMAR)	0.97 ± 0.21	0.85 ± 0.16	2.36, 0.38	0.77 ± 0.23	5.87, 0.0001	0.71 ± 0.19	5.33, 0.0002
RS (dB)	13.78 ± 2.67	15.08 ± 2.32	5.95, < 0.0001	15.42 ± 2.19	6.17, < 0.0001	15.51 ± 2.27	6.34, < 0.0001
Group3 (14 eyes)							
IS/OS defect (μm)	4689.08 ± 978.69	3257.98 ± 892.75	4.64, 0.0005	2671.43 ± 985.54	6.15, < 0.0001	2357.98 ± 924.54	7.07, < 0.0001
BCVA (LogMAR)	1.65 ± 0.44	1.29 ± 0.39	2.75, 0. 17	1.05 ± 0.23	5.10, 0.0002	0.91 ± 0.20	6.57, < 0.0001
RS (dB)	9.07 ± 2.07	11.59 ± 2.45	3.96, 0.002	12.46 ± 2.45	5.21, 0.0002	12.69 ± 2.46	5.47, 0.0001

*BCVA* best-corrected visual acuity, *IS/OS* photoreceptor inner segment and outer segment, *logMAR* logarithm of minimal angle of resolution, *RS* retinal sensitivity. *Student paired *t*-test; p value represents the difference between after and before surgery. P < 0.05 was considered statistically significant

### Functional results

The mean logMAR BCVA of all patients was 1.09 ± 0.53 before surgery and 0.67 ± 0.29 at 9 months after surgery (p < 0.0001). Moreover, the mean BCVA improved significantly after surgery at all follow-ups in each group. The mean BVCA in Group 1 was 0.71 ± 0.33 before surgery and 0.43 ± 0.24 at 9 months after surgery (p < 0.0001). Before surgery, the mean BCVA of Group 2 was 0.97 ± 0.21, whereas this value 9 months after surgery was 0.71 ± 0.19 (p = 0.0002). In Group 3, the mean BCVA values before surgery and 9 months after surgery were 1.65 ± 0.44 and 0.91 ± 0.20 (p < 0.0001), respectively.

The mean RS of all patients was 13.23 ± 3.65 before surgery and 15.43 ± 2.86 at 9 months after surgery (p < 0.0001). The mean RS of Group 1 was 16.27 ± 1.17 dB before surgery and 17.62 ± 1.17 dB at 9 months after surgery (p < 0.0001). Before surgery, the mean RS of Group 2 was 13.78 ± 2.67 dB, whereas at 9 months after surgery, this value was 15.51 ± 2.27 (p < 0.0001). In Group 3, before surgery and 9 months after surgery, the mean RS values were 9.07 ± 2.07 and 12.69 ± 2.46 (p < 0.0001), respectively, and this difference was statistically significant (Table 2).

### Closure Rates

At 9 months after surgery, 11 of 43 (25.6 %) eyes achieved IS/OS junction integrity; 9 (81.8 %) of these 11 eyes belonged to Group 1, 2 (18.2 %) belonged to Group 2. The closure rates were 52.9 % (9 of 17 eyes) in Group 1, 16.7 % (2 of 12 eyes) in Group 2 and 0 in Group 3.

### Correlation between preoperative anatomy and postoperative function

The preoperative IS/OS defect was significantly correlated with the postoperative BCVA and retinal sensitivity at 1 month after surgery (r = 0.71, p < 0.0001). The same result was also observed at 3 and 9 months after surgery (Table 3).

**Table 3:** Regression Analysis Between IS/OS Defect Before Surgery and BCVA, RS Before and After Surgery

Outcomes Parameters	BCVA (logMAR) before and after surgery								Retinal Sensitivity (dB) before and after surgery							
	Before		1 month		3 months		9 months		Before		1 month		3 months		9 months	
	r, p value		r, p value*		r, p value*		r, p value*		r, p value*		r, p value*		r, p value*		r, p value*	
IS/OS defect (μm) before surgery	0.89	<0.0001	0.74	<0.0001	0.79	<0.0001	0.73	<0.0001	0.70	<0.0001	0.48	<0.0001	0.44	<0.0001	0.43	<0.0001
Age		0.76		0.28		0.88		0.77		0.85		0.85		0.76		0.69
Gender		0.82		0.49		0.23		0.25		0.35		0.18		0.11		0.13
Eye		0.11		0.95		0.56		0.26		0.53		0.79		0.52		0.63
Cataract surgery		0.33		0.94		0.38		0.49		0.23		0.43		0.46		0.38
Axial length		0.56		0.21		0.42		0.82		0.35		0.81		0.62		0.52

*BCVA* best-corrected visual acuity, *IS/OS* photoreceptor inner segment and outer segment, *logMAR* logarithm of minimal angle of resolution, *RS* retinal sensitivity. *Multiple linear regression analysis was used to evaluate correlation between IS/OS defect before surgery and BCVA, RS before and after surgery, adjusted with age, gender, eye, cataract surgery and axial length. r value represents adjusted R square. P < 0.05 was considered statistically significant

## Discussion

OCT has recently made it possible to explore changes in ocular layers as axial progresses and the globe is stretched. These findings consist of dehiscence of retinal layers known as retinoschisis, tractional ILM detachment, macular holes (lamellar and full thickness), posterior retinal detachment and choroidal neovascular membranes [10]. According to these OCT findings, patients in this study were separated into 3 groups.

This study showed that HMMH eyes without subretinal fluid (Groups 1 and 2) obtained better postoperative anatomical and functional outcomes than eyes with subretinal fluid (Group 3). In addition, the HMMH patients with ERM traction, retinoschisis (Group 1) regained more visual function than patients with full-thickness macular holes (Group 2). This result suggests that subretinal fluid represents an advanced damage stage. Other research groups have also reported this observation [4–8]. In our study, Group 1 through 3 approximated HMMH at varying severities stage in its natural course. Indeed, preoperative IS/OS defects, BCVA and RS all differed significantly among the three groups, providing clinical significance for the grouping of patients performed in our study.

The integrity of inner and outer segments of photoreceptors (IS/OS) is closely related to visual recovery after successful MH closure surgery [11–15]. Visual acuity is generally used as a gold standard to indicate visual function, although it represents only one aspect. In this study, microperimetry was applied to quantify foveal and perifoveal retinal sensitivity and correlate these results to the microstructural findings in HMMH [16, 17]. Specifically, we used the IS/OS defect as an anatomical parameter and BCVA and retinal sensitivity (RS) as functional parameters to evaluate the relationship between microstructure and function in the HMMH eyes that underwent surgery.

We found that in all 3 groups, the IS/OS defect decreased significantly as time progressed, indicating a potential regeneration of photoreceptors. Similarly, BCVA and RS measurements gradually reached significant improvement after surgery. Interestingly, the preoperative IS/OS defect was significantly correlated with both the preoperative and postoperative BCVA and RS values (Table 3). Although great care must be taken with small patient cohorts, our study revealed the potential prognostic value of the size of the IS/OS defect in conjunction with other known factors (e.g., retinal or RPE atrophy) and the preoperative diameters of macular holes.

Furthermore, 52.9 % (9 of 17 eyes) eyes in Group 1 approached postoperative closure, only 16.7 % (2 of 12 eyes) in Group 2 and 0 in Group 3 at 9 months after surgery. The minimum preoperative diameter of IS/OS defect in each group was 305.88 μm in Group 1, 1258.82 μm in Group 2, and 2317.64 μm in Group 3. As this parameter has showed a potential prognostic value, this result explained the significant differences in postoperative closure rates between groups.

Subretinal fluid is a generally regarded as a negatively prognostic complication of HMMH, because, in theory, chronic fluid may aggravate damage to photoreceptors. Assuming that subretinal fluid is a severity-dependent complication in the evolution of HMMH, it is even more important to perform effective surgery at a less damage stage for optimal postoperative functional recovery and restoration. Our study suggests that the appearance of ERM traction and consequent retinoschisis without subretinal fluid in HMMH, indicates a better prognosis for surgical intervention. Although some patients may show no subjective symptoms for years at this stage [18–20], visual acuity impairment should indicate surgery in the affected eye.

Based on its low complication rate, transconjunctival 23-gauge (23G) pars plana vitrectomy, as performed in our cases, was an effective treatment for HMMH. Nonetheless, our study lacks long-term results exceeding 9 months. Indeed, vitrectomy in myopic cases is associated with a postoperative risk for retinal detachment and subsequent visual loss [21], which we fortunately did not observe within these 9 months. Generally, MH closure is less frequently observed following vitrectomy in HMMH compared to emmetropic cases [22]. These less favorable results may be explained by the presence of persistent macular traction because of continued elongation and staphyloma formation in pathologic myopia. Based on this hypothesis, other groups have suggested that an additional procedure, such as an episcleral macular buckle, may be useful to counteract the posterior traction [21, 23]. Although a variety of procedures are available for HMMH, there is still no unified standard treatment method. Therefore, additional longer-term studies are required.

ICG toxicity and damage to the retina has been reported in in vitro and in vivo studies, and following macular surgery. Toxic effects can occur to retinal glial cells, to the nerve fiber layer, to retinal ganglion cells, and to the optic nerve. ICG at concentrations higher than 1.25 % or application of the dye in air are very likely causing retinal damage [24]. In this study, a concentration of was applied in every case with a less than 30 seconds dyeing time to minimize the ICG toxicity as far as possible.

Our study has a number of limitations. First, none of the eyes in Group 3 and very few in Group 2 closed, whereas the postoperative BCVA were all significantly improved. This may result from the underestimation of the preoperative visual acuity due to cataract. The improved paracentral vision thanks to the regeneration of IS/OS structure as time progressed could also be a part of the explanation. Second, ICG toxicity and damage to the retina has been reported in in vitro and in vivo studies, and following macular surgery. Toxic effects can occur to retinal glial cells, to the nerve fiber layer, to retinal ganglion cells, and to the optic nerve. ICG at concentrations higher than 1.25 % or application of the dye in air are very likely causing retinal damage [24]. In this study, a concentration of was applied in every case with a less than 30 seconds dyeing time to minimize the ICG toxicity as far as possible. Third, this study lacks long-term results exceeding 9 months. Further prospective, long-term study would be needed for detection of further anatomical and functional changes.

## Conclusions

In conclusion, pars plana vitrectomy combined with ILM peeling and gas tamponade results in limited functional outcomes in patients with HMMH. In HMMH, the appearance of subretinal fluid indicates a worse prognosis for surgical intervention.

## Abbreviations

BCVA: best-corrected visual acuity
HMMH: high myopia macular hole
ICG: Indocyanine green
ILM: internal limiting membrane
IS/OS: photoreceptor inner and outer segments
MTM: myopic traction maculopathy
MP1: Microperimetry 1
OCT: optical coherence tomography
RS: retinal sensitivity
FTMH: full-thickness macular hole
SD-OCT: spectral domain OCT
ERM: epiretinal membrane
